# Combining metabolomics and machine learning to discover biomarkers for early-stage breast cancer diagnosis

**DOI:** 10.1371/journal.pone.0311810

**Published:** 2024-10-21

**Authors:** Nguyen Ky Anh, Anbok Lee, Nguyen Ky Phat, Nguyen Thi Hai Yen, Nguyen Quang Thu, Nguyen Tran Nam Tien, Ho-Sook Kim, Tae Hyun Kim, Dong Hyun Kim, Hee-Yeon Kim, Nguyen Phuoc Long

**Affiliations:** 1 Faculty of Pharmacy, Ton Duc Thang University, Ho Chi Minh City, Vietnam; 2 Department of Pharmacology and PharmacoGenomics Research Center, Inje University College of Medicine, Busan, Republic of Korea; 3 Department of Surgery, Chung-Ang University Gwangmyeong Hospital, Chung-Ang University College of Medicine, Gyeonggi-do, Republic of Korea; 4 Department of Surgery, Busan Paik Hospital, Inje University College of Medicine, Busan, Republic of Korea; University of California Riverside, UNITED STATES OF AMERICA

## Abstract

There is an urgent need for better biomarkers for the detection of early-stage breast cancer. Utilizing untargeted metabolomics and lipidomics in conjunction with advanced data mining approaches for metabolism-centric biomarker discovery and validation may enhance the identification and validation of novel biomarkers for breast cancer screening. In this study, we employed a multimodal omics approach to identify and validate potential biomarkers capable of differentiating between patients with breast cancer and those with benign tumors. Our findings indicated that ether-linked phosphatidylcholine exhibited a significant difference between invasive ductal carcinoma and benign tumors, including cases with inconsistent mammography results. We observed alterations in numerous lipid species, including sphingomyelin, triacylglycerol, and free fatty acids, in the breast cancer group. Furthermore, we identified several dysregulated hydrophilic metabolites in breast cancer, such as glutamate, glycochenodeoxycholate, and dimethyluric acid. Through robust multivariate receiver operating characteristic analysis utilizing machine learning models, either linear support vector machines or random forest models, we successfully distinguished between cancerous and benign cases with promising outcomes. These results emphasize the potential of metabolic biomarkers to complement other criteria in breast cancer screening. Future studies are essential to further validate the metabolic biomarkers identified in our study and to develop assays for clinical applications.

## Introduction

Breast cancer (BC) ranks among the most prevalent malignant neoplasms in women. The World Health Organization (WHO) reported alarming estimates of over 2.3 million BC diagnoses and 685,000 BC fatalities in 2020 [1]. Particularly concerning fact is that BC is the foremost cause of cancer-related deaths in women under the age of 45 years [2]. The elevated mortality rate associated with BC in numerous countries can be attributed to inadequacies in screening, early detection, and diagnosis [1]. The importance of screening and detecting BC at an initial stage cannot be overstated, as this is pivotal in enhancing treatment efficacy and decreasing mortality. Approaches ranging from imaging techniques to molecular biomarkers [3, 4], not only strive for precise diagnosis but also aim to classify BC subtypes, thereby guiding oncological decision-making [5]. For example, BC can be classified as preinvasive (ductal carcinoma in situ and lobular carcinoma in situ) and invasive (ductal carcinoma and lobular carcinoma) based on histological information, or molecular subtypes such as luminal A, luminal B and triple-negative BC based on immunohistochemistry information [6].

In the past several decades, mammography has emerged as a principal tool for BC screening and has contributed to a decline in mortality rates [7]. Furthermore, the incorporation of artificial intelligence and machine learning is garnering attention because of their potential to improve the accuracy of BC diagnosis based on mammography images [8]. According to the Breast Imaging Reporting and Data System (BI-RADS) lexicon, mammography results can be divided into 7 categories. Category 0 stands for incomplete information. Categories 1 to 3 are related to cancer negative, benign or probably benign. Category 4 represents cases with a likelihood of BC. Category 5 and 6 indicated highly suggestive and biopsy-proven of malignancy, respectively [9]. Despite proven clinical merits, this technique is hampered by a high false-positive rate, which can contribute to misdiagnosis [7]. Moreover, conventional classification methods do not adequately address the diverse clinical trajectories of individual cancer cases [5]. Consequently, molecular classifications employing cutting-edge, high-throughput technologies, such as multi-omics, are under investigation for their potential to enhance BC diagnosis [5].

Blood-based biomarkers have shown promise in the early detection and diagnosis of BC. Cancer antigen 15–3 (MUC-1 antigen) and carcinoembryonic antigen are two serum biomarkers that have been applied in clinical settings [10, 11]. However, the limited sensitivity and selectivity of these markers can result in misdiagnosis [10]. This underscores the need to identify novel biomarkers with high sensitivity and selectivity to improve BC diagnosis.

Metabolic rewiring is a hallmark of cancer and is closely associated with tumor initiation, progression, metastasis, and resistance to antineoplastic drugs [12]. Untargeted metabolomics and lipidomics utilizing liquid chromatography—tandem mass spectrometry (LC-MS/MS) have demonstrated immense potential in the discovery of novel biomarkers and the generation of hypotheses concerning metabolic alterations [13]. Accordingly, several studies have explored the metabolic and lipid profiles of BC patients using high-throughput LC-MS/MS [14–16]. For example, L-octanoylcarnitine, 5-oxoproline, hypoxanthine, and docosahexaenoic acid have been identified as potential plasma biomarkers for BC diagnosis [16]. Moreover, L-arginine and arachidonic acid could be used for both detecting BC and predicting the efficacy of trastuzumab [17]. In addition, lipids play a significant role in cell signaling processes, which are linked to membrane properties, metabolism, and the invasive and metastatic behavior of tumor cells [14, 18]. Therefore, metabolomics and lipidomics research is indispensable for the early detection, accurate diagnosis, prognosis, and treatment of BC [16, 19].

This study aimed to employ a multimodal omics approach in conjunction with machine learning models to identify and validate potential endogenous biomarkers that can differentiate the metabolic and lipid profiles of BC versus benign patients. Our findings highlighted several metabolites and lipids, particularly ether-linked phosphatidylcholine (PC(O-)), which exhibited significant alterations in BC compared with benign tumors. Additionally, we identified a metabolism-centric biosignature that exhibited good performance in cases where mammography yielded suboptimal results. The insights of this study will potentially serve as a foundation for the development of supplementary tools enhancing the effectiveness of mammography in the screening and early diagnosis of BC.

## Materials and methods

### Clinical samples and ethical approval

Patients recruited between January 1^st^, 2019, and July 30^th^, 2022, who had available clinical data along with plasma samples stored in the Inje University College of Medicine Biobank were included in this study. All participants provided written informed consent for the use of their clinical information and plasma samples for research purpose of the biobank. The study was conducted with the approval of the Institutional Review Board of Inje University College of Medicine Busan Paik Hospital (IRB No. 2022-08-052). The specimens were acquired from the biobank after the IRB approval on September 16^th^, 2022. The study used information distributed from the biobank and identifiable information was not obtained.

### Clinical characteristics of the patients and study design

**Fig 1:**
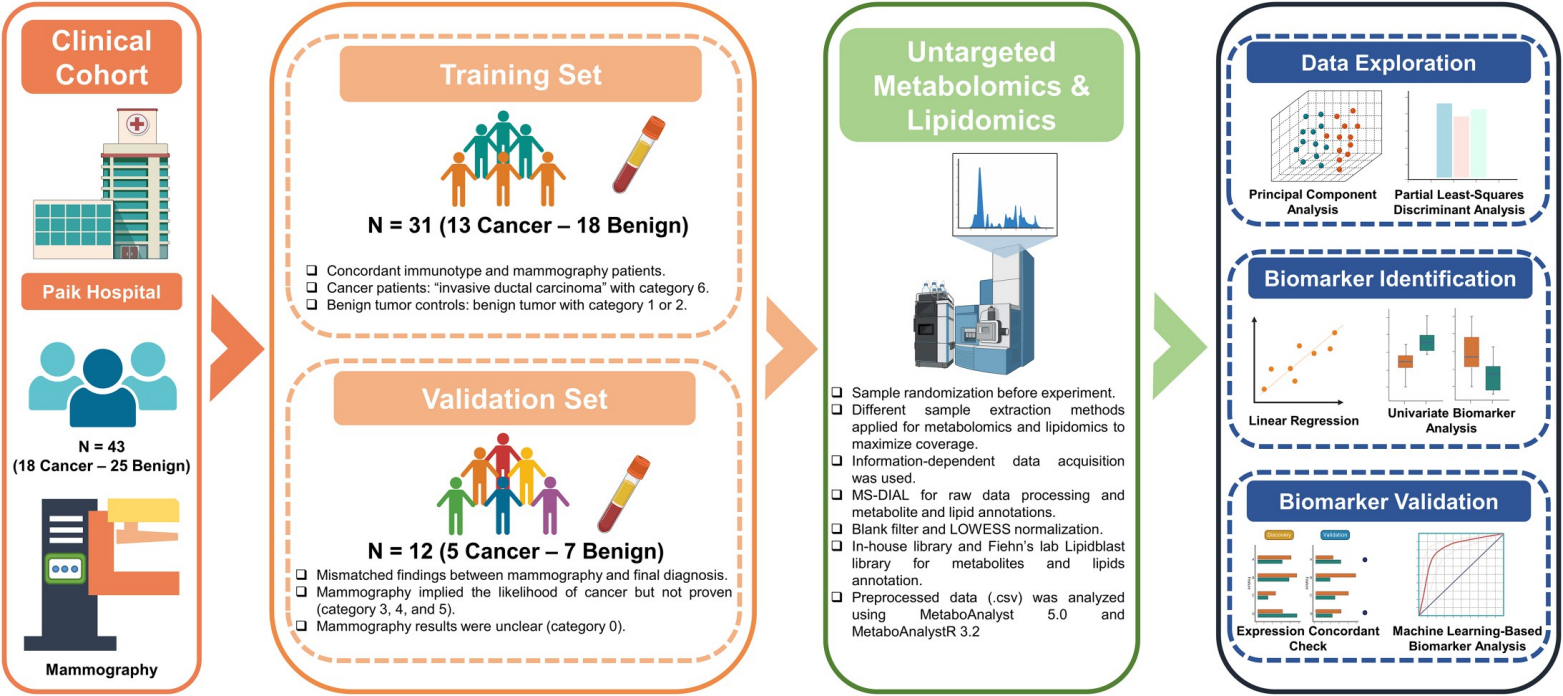
Schematic diagram representing the study design and computational workflow.

The design and workflow of the study are illustrated in Fig 1. For the training set, aimed at the discovery of potential biomarkers, patients who had concordant preoperative mammography (Mammography BI-RADS Assessment Categories) findings and immunohistochemistry results obtained from biopsy procedures were included [20]. Specifically, the cancer group comprised 13 patients diagnosed with invasive ductal carcinoma who had category 6 mammography findings. The benign group included 18 patients with benign tumors and mammography findings categorized as 1 or 2. For the validation set, patients with discordant findings between the mammography category and immunohistochemistry results were included. This set consisted of five patients with invasive ductal carcinoma and seven cases with benign tumors. The available clinical characteristics provided by the biobank are presented in Table 1.

**Table 1:** The clinical characteristics of the patients.

Training set				
			Cancer group (N = 13)	Benign group (N = 18)
Age, year, mean (range)			47.5 ± 5.8 (37–55)	42.4 ± 12.2 (20–68)
BMI, kg/m^2^, mean (range)			22.16 ± 1.37 (19.37–24.31)	22.82 ± 3.41 (18.86–31.75)
Mammography, n (%)		Category 0	-	-
		Category 1	-	12 (66.7%)
		Category 2	-	6 (33.3%)
		Category 3	-	-
		Category 4A	-	-
		Category 4B	-	-
		Category 4C	-	-
		Category 5	-	-
		Category 6	13 (100.0%)	-
IHC, n (%)		M9020/0	-	7 (38.9%)
		M8500/3	13 (100.0%)	-
		Unknown	-	11 (61.1%)
Molecular subtype		Luminal A	2 (15.4%)	-
		Luminal B	4 (30.7%)	-
		Luminal B & HER2 positive	3 (23.1%)	-
		HER2 positive	1 (7.7%)	-
		Triple negative	3 (23.1%)	-
Final diagnosis, n (%)	Invasive ductal carcinoma		13 (100.0%)	-
	Benign	Fibroadenoma	-	2 (11.1%)
		Fibrocystic change	-	3 (16.7%)
		Fibroepithelial tumor	-	9 (50.0%)
		Intraductal papilloma with epithelial hyperplasia	-	1 (5.6%)
		Mature adipose tissue	-	1 (5.6%)
		Paraffinoma	-	2 (11.1%)
Stage (TNM categories), n (%)	T	T0	1 (7.7%)	-
		T1a	1 (7.7%)	-
		T1b	1 (7.7%)	-
		T1c	5 (38.5%)	-
		T2	4 (30.8%)	-
		Tis	1 (7.7%)	-
		Unclassified	-	18 (100%)
	N	N0	7 (53.9%)	-
		N1a	5 (38.5%)	-
		Nx	1 (7.7%)	-
		Unclassified	-	18 (100%)
Chemotherapy received, n (%)			10 (76.9%)	-
Test set				
			Cancer group (N = 5)	Benign group (N = 7)
Age, year, mean (range)			51.4 ± 7.2 (46–63)	46.1 ± 7.4 (37–55)
BMI, kg/m^2^, mean (range)			24.59 ± 5.14 (18.68–31.89)	24.03 ± 3.58 (18.59–29.52)
Mammography, n (%)		Category 0	1 (20.0%)	2 (28.6%)
		Category 1	-	-
		Category 2	3 (60.0%)	-
		Category 3	-	3 (42.8%)
		Category 4A	-	1 (14.3%)
		Category 4B	1 (20.0%)	-
		Category 4C	-	-
		Category 5	-	-
		Category 6	-	1 (14.3%)
IHC, n (%)		M9020/0	-	2 (28.6%)
		M8500/3	5 (100.0%)	-
		Unknown	-	5 (71.4%)
Molecular subtype		Luminal A	2 (40.0%)	-
		Luminal B	1 (20.0%)	-
		Triple negative	2 (40.0%)	-
Final diagnosis, n (%)	Invasive ductal carcinoma		5 (100.0%)	-
	Benign	Fibroadenoma	-	2 (28.6%)
		Fibrocystic change	-	2 (28.6%)
		Follicular lymphoma	-	1 (14.3%)
		Phyllodes tumor	-	2 (28.6%)
Stage (TNM categories), n (%)	T	T0	-	-
		T1a	1 (20.0%)	-
		T1b	1 (20.0%)	-
		T1c	2 (40.0%)	-
		T2	1 (20.0%)	-
		Tis	-	-
		Unclassified	-	7 (100%)
	N	N0	5 (100%)	-
		N1a	-	-
		Nx	-	-
		Unclassified	-	7 (100%)
Chemotherapy received, n (%)			-	-

Abbreviations: BMI, Body Mass Index; IHC, immunohistochemistry; T, Tumor; N, Node; M, Metastasis.

### Reagents and chemicals

LC-MS-grade solvents including water, acetonitrile, methanol (MeOH), and isopropanol were sourced from Merck KGaA (Darmstadt, Germany). Formic acid, ammonium formate, ammonium acetate, methyl *tert*-butyl ether (MTBE), and toluene were procured from Sigma-Aldrich (St. Louis, Missouri, USA). Internal standards (IS) for metabolomics, such as acetyl-L-carnitine-(*N*-methyl-d3), L-phenyl-d5-alanine, L-tryptophan-(indole-d5), leucine enkephalin, SM(d18:1/15:0)-d9, and cholic acid-2,2,3,4,4-d5, were also obtained from Sigma-Aldrich (St. Louis, Missouri, USA). For lipidomics analysis, the LIPIDOMIX^®^ Mass Spec Standard and Deuterated Ceramide Mass Spec Standard were acquired from Avanti Polar Lipids (Alabama, USA). The UPLC ACQUITY ethylene bridged hybrid (BEH) C18 column (100 mm × 2.1 mm, 1.7 μm) was used for metabolomics, while the UPLC ACQUITY BEH C18 column (50 mm × 2.1 mm; 1.7 μm), paired with a UPLC BEH C18 VanGuard pre-column (5 mm × 2.1 mm; 1.7 μm) (Waters, Milford, MA, USA), was employed for lipidomics analysis.

### Sample preparation

For untargeted metabolomics, we employed the extraction protocol outlined in our previous study [21]. Samples were stored at -80°C prior to any experiment. Initially, 50 μL of the plasma samples were thawed on ice for 30 min and then briefly vortexed for 10 s. Next, 150 μL MeOH at −20°C, containing premixed IS was added to the plasma. The mixtures were vortexed for 30 s and centrifuged at 14,000 *rcf* and 4°C for 2 min. Thereafter, 150 μL of the supernatant was transferred to a new tube and evaporated completely under a stream of nitrogen gas at room temperature. For LC-MS/MS analysis, the dried extracts were reconstituted in 200 μL 50% MeOH and centrifuged for 2 min at 14,000 *rcf* and 4°C. Subsequently, 100 μL of the supernatant from each sample was allocated for analysis, while the remaining 50 μL was used to create pooled quality control (QC) samples.

For lipidomics, we utilized an MTBE-based biphasic extraction method, which was based on previously established protocols with minor modifications [21, 22]. Briefly, 5 μL of each lipid IS mix was spiked into every sample (50 μL) after thawing for approximately 30 min, followed by brief vortexing. The mixtures were then incubated on ice for 20 min, with intermittent vortexing. Subsequently, 300 μL of MeOH and 1,000 μL MTBE, both at −20°C were added to the samples. The mixtures were vortexed for 10 s and then shaken for 20 min at 1,200 *rpm* and 4°C. Following this, 250 μL water was added to the samples, and the samples were vortexed for 20 s. The samples were then centrifuged at 14,000 *rcf* and 4°C for 2 min, after which 500 μL of the upper phase was collected and transferred to a new tube. The lipid extracts were dried under a stream of nitrogen gas at room temperature and stored at −80°C until analysis. For reconstitution, the dried extracts were dissolved in 200 μL of a MeOH/toluene mixture (9:1, *v/v*). QC samples were generated by pooling 50 μL of each sample, and the rest of the sample was used for untargeted lipidomics analysis.

### Data acquisition using LC-MS/MS

Data acquisition for both metabolomics and lipidomics analyses was performed using the Shimadzu Nexera LC (Kyoto, Japan) system coupled with the X500R Quadrupole Time-of-Flight mass spectrometer (SCIEX, MA, USA). The autosampler temperature was maintained at 4°C. For untargeted metabolomics, the gradient method, injection volumes, data acquisition, and MS/MS settings were selected in accordance with our previously established protocol [21]. For lipidomics, we utilized the rapid LC method for untargeted lipidomics as described by Cajka *et al*. [23]. The injection volume was set at 1 μL for the positive ion (ESI+) mode and 3 μL for the negative ion (ESI-) mode. Data Dependent Acquisition was employed for data acquisition, using the same MS/MS parameters as in our prior study [21]. Mass calibration was executed after every four injections for metabolomics and after eight injections for lipidomics via the X500R’s calibrant delivery system to ensure the quality of analysis.

### Data processing and treatment

The raw mass spectrometry data files (.wiff) were processed using MS-DIAL version 4.9.0. The parameters for MS-DIAL were based on our previous study [21]. For metabolomics, the retention time was corrected using internal standards. Statistically significant hydrophilic metabolites were identified based on the established in-house library [21], the public MS-DIAL libraries, and IRCCS Istituto Giannina Gaslini-Mass Spectra Library [24]. The data were subsequently normalized using a locally weighted scatterplot smoothing (LOWESS) algorithm and the IS-normalization method. For lipidomics, lipids were annotated using MS-DIAL built-in library and Fiehn’s lab lipidomics library [25, 26]. The lipidomics data were also LOWESS-normalized.

### Exploratory data analysis

The aligned data exported from MS-DIAL were analyzed using the MetaboAnalyst 5.0 platform and the MetaboAnalystR package version 3.2.0 to retain only the features with a missing rate of 50% or less [27, 28]. Missing values in these features were then imputed using the k-nearest neighbors algorithm. Features displaying a relative standard deviation exceeding 25% in the QC samples were excluded. Both metabolomic and lipidomic profiles were visualized employing principal component analysis (PCA), wherein the data were log-transformed and Pareto-scaled. To explore distinctions between the cancer and benign groups, a partial least squares—discriminant analysis (PLS-DA) was conducted. The performance of the PLS-DA model was evaluated via a five-fold cross-validation method, with Q^2^ used to determine the optimal model. Unless specified otherwise, data visualization was performed using the ggplot2 package (version 3.4.1) in R version 4.2.2.

### Statistical analysis

In this study, the training set was used to identify differential molecules (DMs) in BC and potential biomarkers differentiating between the cancer and benign groups. Linear models incorporating age and BMI adjustments (for DMs) and classical univariate receiver operating characteristic (ROC) curve analysis were conducted using MetaboAnalyst 5.0. The thresholds for statistical significance in the linear models were a p-value of 0.05 and a false discovery rate (FDR) of 0.25. Additionally, features with an area under the ROC curve (AUC) ≥ 0.7 and p-value < 0.05 were chosen as potential biomarker candidates. All potential biomarker candidates identified in the training set underwent validation using the validation set. Biomarkers that showed consistency between the training and validation sets were subjected to classical univariate ROC analysis. The AUC and p-value obtained from the models were used to assess the ability of the biomarkers to differentiate cancer from benign patients. Candidates demonstrating robust performance and consistent expression between the training and validation set were selected for univariate ROC analysis using the validation data. Subsequently, the best performers were chosen to create a single biosignature. The diagnostic potential of this biosignature was then assessed by a multivariate machine learning-based ROC model using the validation set. Linear support vector machine (SVM) and random forest algorithms, in conjunction with age and BMI as covariates, were implemented.

## Results

### Data exploration revealed subtle differences in plasma metabolic profiles between cancer and benign groups

**Fig 2:**
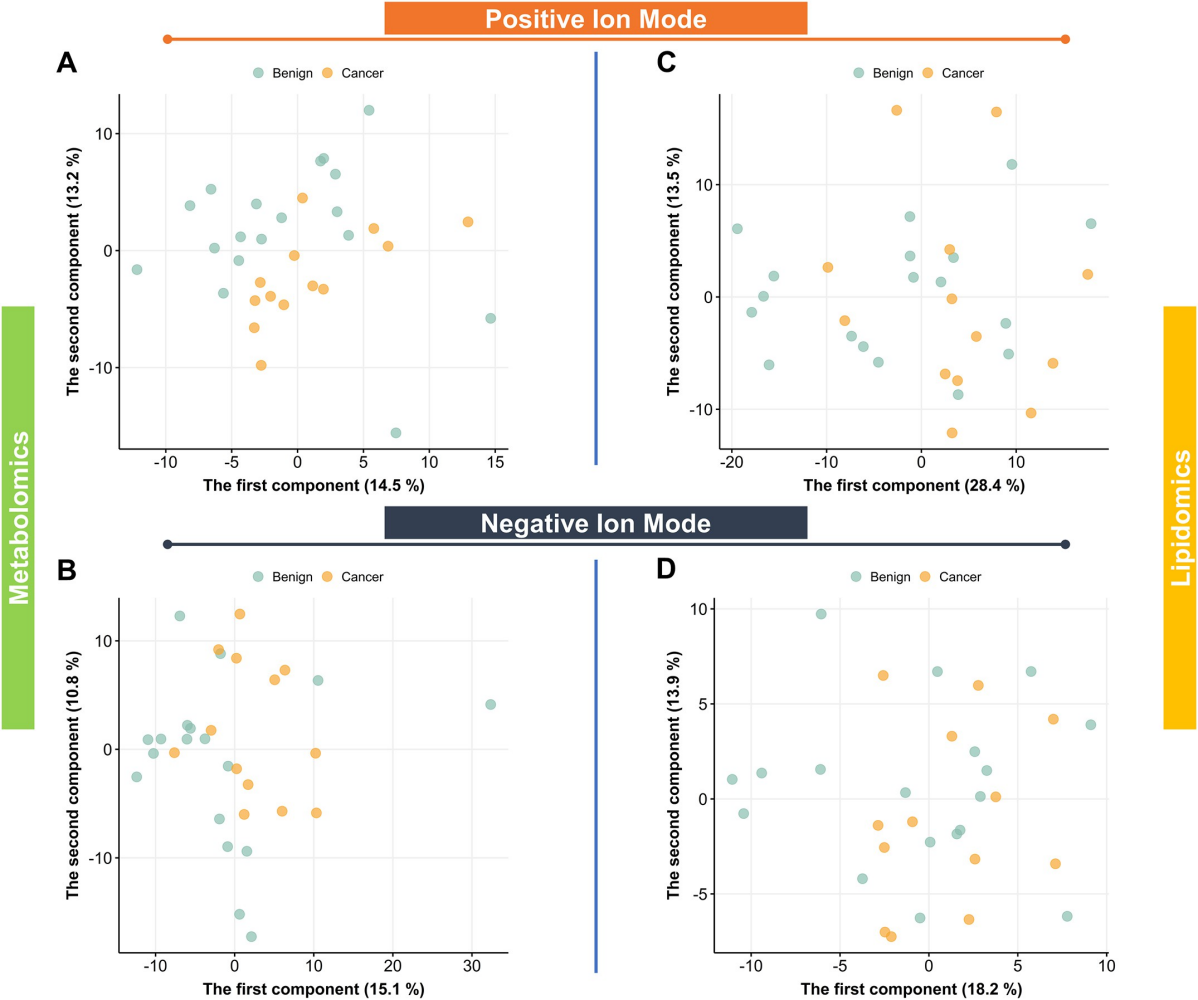
PCA scores plot visualizing metabolomic and lipidomic profiles in the breast cancer and benign tumor control groups. A. Metabolomics positive ion mode. B. Metabolomics negative ion mode. C. Lipidomics positive ion mode. D. Lipidomics negative ion mode.

PCA was performed on the metabolomics data to examine sample trends without considering the origins of the samples. In the PCA scores plots between patient’s and QC samples, the QC samples clustered indicating repeatability and satisfactory data acquisition process (S1A, S1B Fig in S1 File). The PCA scores plot without QC samples of the metabolomics data in ESI+ mode hinted at a slight distinction between the cancer and benign groups, whereas the PCA scores plot of ESI- mode did not display any apparent separation (Fig 2A and 2B). PCA was also applied to the lipidomics data in both the ESI+ and ESI- modes. The PCA scores plots with QC samples showed clustering of QC samples similar to metabolomics analysis (S1C, S1D Fig in S1 File). The PCA scores plot of clinical samples in ESI+ mode exhibited a general overlap between the cancer and benign groups (Fig 2C). In line with the results from ESI+ mode, no significant separation was evident between the cancer and benign groups in the PCA scores plot of ESI- mode (Fig 2D). Notably, the variance explained by PC1 and PC2 was considerably below 50%, indicating that the relationship among features was complex, and a linear model utilizing the complete profile might not effectively capture the biological variance between the two groups.

**Fig 3:**
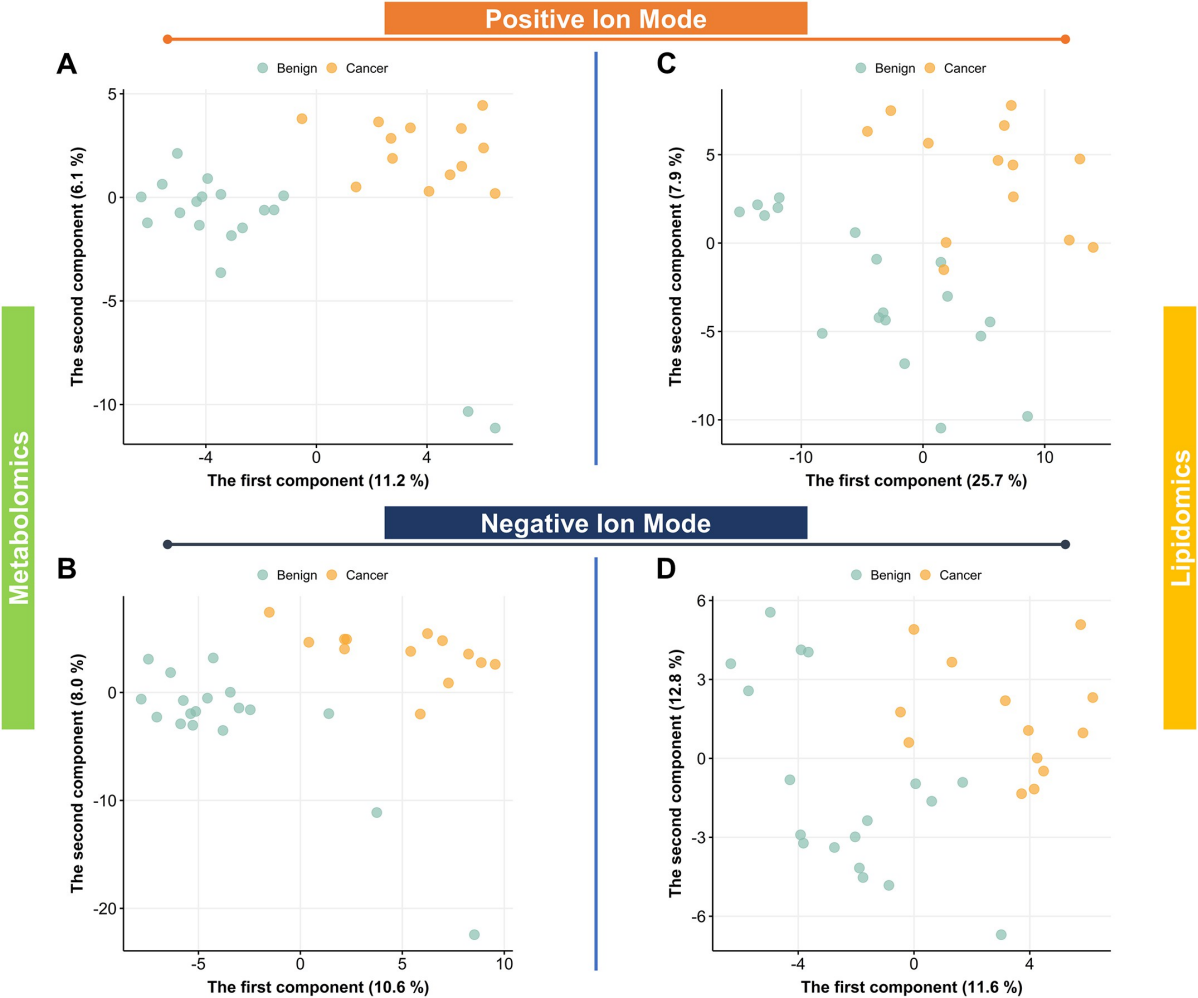
PLS-DA scores plot visualizing metabolomic and lipidomic profiles in the breast cancer and benign tumor control groups. A. Metabolomics positive ion mode. B. Metabolomics negative ion mode. C. Lipidomics positive ion mode. D. Lipidomics negative ion mode.

Subsequently, PLS-DA was conducted using the metabolic profiles to discern differences between the cancer and benign groups. Analysis using the ESI+ mode metabolomics data showed that PLS-DA could not efficiently distinguish the two groups (accuracy = 0.757, R^2^ = 0.944, Q^2^ < 0, Fig 3A). In accordance with the ESI+ mode, PLS-DA of ESI- mode data failed to reliably differentiate the two groups (accuracy = 0.600, R^2^ = 0.997, Q^2^ < 0, Fig 3B). The models demonstrated unsatisfactory predictive performance, as indicated by the negative Q^2^ values. When analyzing lipidomics data, PLS-DA scores plot in ESI+ mode revealed some separation in lipid profiles between the cancer and benign groups but with limited predictive accuracy (accuracy = 0.803, R^2^ = 0.982, Q^2^ = 0.246, Fig 3C), as did PLS-DA in ESI- mode (accuracy = 0.672, R^2^ = 0.573, Q^2^ = 0.111, Fig 3D). The results suggest that, similar to metabolomics analysis, PLS-DA in the lipidomics analysis also possessed limited predictive ability (Q^2^ < 0.4).

In summary, the data exploration implied subtle differences in plasma metabolic profiles between the cancer and benign groups. However, both PCA and PLS-DA revealed that the distinctions were not pronounced, and the predictive performance was limited.

### Univariate biomarker analysis identified potential biomarker candidates

A linear model, adjusted for age and BMI, was employed to identify molecules with significant differences between the cancer and benign groups. The analysis utilizing the linear model with metabolomics data revealed 86 significant features in ESI+ mode (9 upregulated and 77 downregulated in cancer) and 52 significant features in ESI- mode (30 upregulated and 22 downregulated in cancer), based on p < 0.05. Notably, only the ether-linked lysophosphatidylcholine (LPC) (O-22:2) remained significant after applying an adjusted p-value (FDR < 0.25). Using lipidomics data, the linear model analysis yielded 141 significant features in ESI+ mode (25 upregulated and 116 downregulated in cancer) and 76 significant features in ESI- mode (28 upregulated and 48 downregulated in cancer), with p < 0.05. Among these, 30 lipids in ESI+ mode and 11 in ESI- mode retained significance after adjusting the p-value (FDR < 0.25). The significant lipids were classified into subclasses including LPC(O-), PC(O-), sphingomyelin (SM), and free fatty acids (FA).

The results from the linear model, adjusted for age and BMI, were in harmony with the observations made in the exploratory data analysis, wherein only a handful of metabolites and lipids fulfilled the criteria for statistical significance. Consequently, univariate ROC analysis was employed to further assess the potential of plasma polar metabolites and lipids to distinguish between cancer and benign cases. In the metabolomics analysis, two annotated features in ESI+ mode and six in ESI- mode exhibited an AUC ≥ 0.7 for differentiating between the cancer and benign groups. Among these metabolites, deoxycholic acid glycine conjugate/glycoursodeoxycholic acid and LPC(O-22:2) demonstrated good performance (AUC > 0.8). In the lipidomics data analysis, a total of 53 lipids in ESI+ mode and 17 in ESI- mode achieved an AUC ≥ 0.7 (p < 0.05). Remarkably, PC(O-48:8) and PC(O-42:2) exhibited exceptional performance in differentiating between the cancer and benign groups (AUC > 0.9). Moreover, 14 lipids in ESI+ mode and 5 lipids in ESI- mode achieved an AUC > 0.8.

In summary, the univariate ROC analysis identified 73 potential biomarker candidates in BC (5 of which were detected in both ion modes), while the linear model detected 38 DMs in BC (4 of which were detected in both ion modes). The statistical attributes of these DMs and biomarker candidates are provided in S1 Table in S1 File.

### External validation highlighted the potential of metabolism-centric biomarkers in aiding BC diagnosis

External validation was conducted to evaluate the potential diagnostic abilities of biomarkers identified in the training set, utilizing a distinct dataset. Initially, biomarker candidates with an AUC ≥ 0.7 and p < 0.05 in the univariate ROC analysis were chosen. In instances where candidates were detected in both ion modes, data from the ESI+ mode was selected. Subsequently, the expression direction of the biomarker candidates was compared between the training and validation datasets. A total of 61 candidates that demonstrated consistency in expression trends between both datasets were selected for further validation (S2 Fig in S1 File).

**Fig 4:**
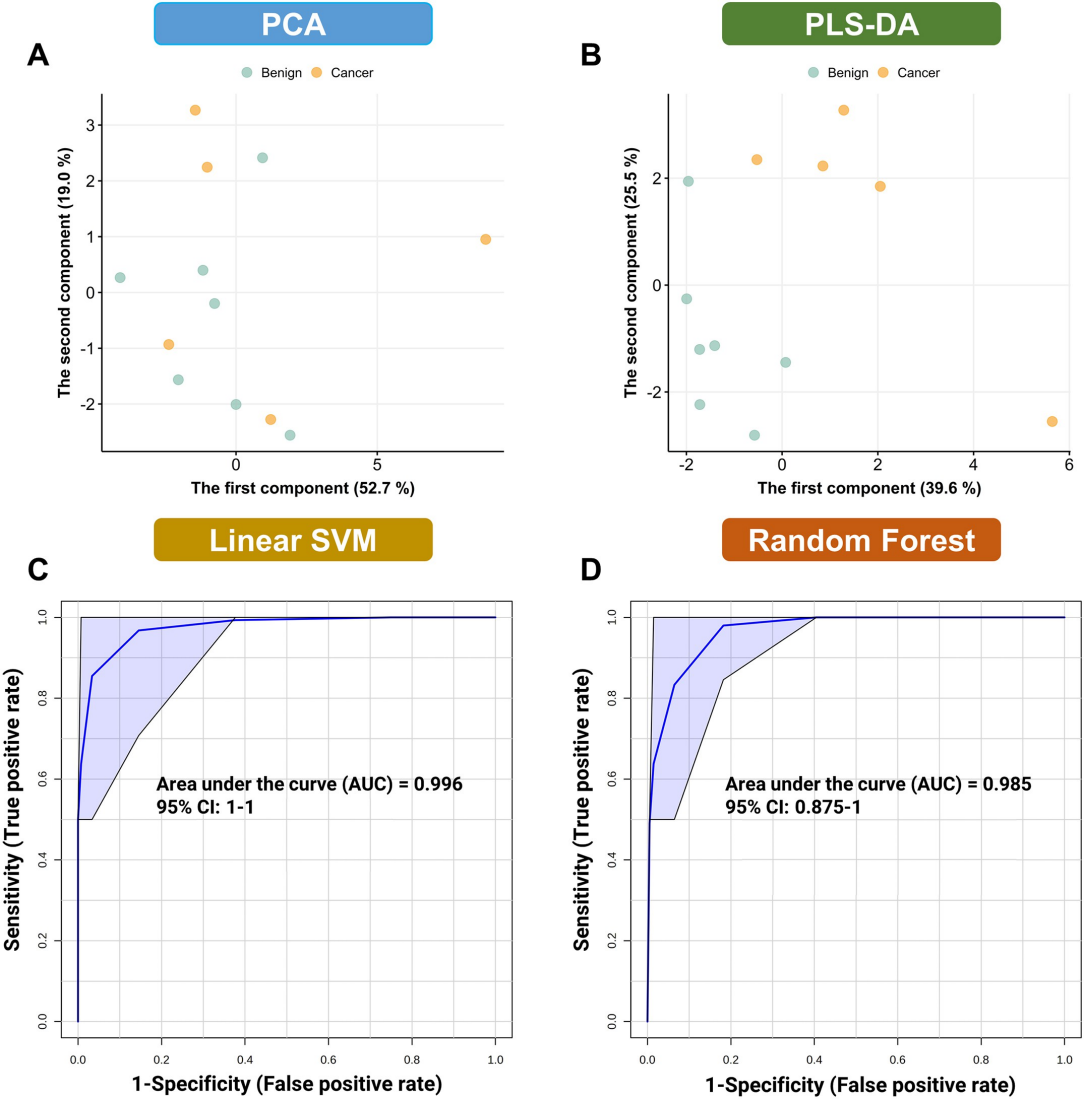
External validation of the metabolism-centric biosignature. A. PCA scores plot. B. PLS-DA scores plot. C. Linear SVM ROC curve. D. Random Forest ROC curve. Abbreviations: CI, Confidence interval.

As part of the exploratory data analysis, PCA and PLS-DA were performed using the biomarker candidates to distinguish between cancer and benign cases within the validation set. Importantly, the PLS-DA scores plot displayed a distinct separation between the two groups along with strong predictive ability (accuracy = 0.933, R^2^ = 0.996, Q^2^ = 0.697, Fig 4A and 4B). This highlights the potential of the chosen biomarker candidates in differentiating between cancer and benign cases.

Additionally, univariate ROC analysis was employed in the validation set to assess the classification performance of the biomarker candidates. Specifically, seven biomarker candidates demonstrated robust performance in differentiating between the cancer and benign groups and were thus chosen to create a composite signature (Table 2). Interestingly, though consistent as a biomarker, PC(O-) did not exhibit as of strong performance in differentiating between cancer and benign groups in the validation set as it did in the training set. This implies that individual biomarkers may not consistently deliver reliable results for precise BC diagnostics.

**Table 2:** The classic univariate ROC analysis on the validation set of significant biomarker candidates.

ID	Analyte	AUC	p-value
1	Dimethyluric acid	1.000	5.75E-05
2	Paraxanthine	1.000	1.39E-04
3	PC(O-33:2)	0.943	4.61E-03
4	Deoxycholic acid glycine conjugate/Glycoursodeoxycholic acid	0.886	2.49E-02
5	PC(32:2)	0.886	1.45E-02
6	SM(40:2;2O)	0.829	3.49E-02
7	SM(41:2;2O)	0.829	3.90E-02

Abbreviations: AUC, Area Under the Curve; PC, Phosphatidylcholine; PC (O-), Ether-linked Phosphatidylcholine; SM, Sphingomyelin.

Consequently, multivariate machine learning models, namely linear SVM and random forest, incorporating age, BMI, and the refined signature, were employed to classify BC cases in the validation set. Notably, the machine learning model exhibited exceptional performance in distinguishing between cancer and benign cases (Fig 4C and 4D). The multivariate ROC analysis employing linear SVM yielded an AUC of 0.996 (95% CI, 1.000–1.000), while the random forest model yielded an AUC of 0.985 (95% CI, 0.875–1.000).

These outcomes of the multivariate ROC analysis using machine learning models suggest that metabolism-centric biomarkers hold promise for enhancing the accuracy of BC screening and diagnosis.

## Discussion

Early detection of BC is crucial for reducing patient mortality. However, mammography may not be sufficiently effective for the screening and accurate diagnosis of BC due to the inherent molecular heterogeneity of the disease [5]. Moreover, high-throughput technologies have provided multiple opportunities for biomarker discovery and validation, which can significantly enhance BC diagnosis and subtyping [5, 29]. Numerous studies have documented alterations in the metabolome and lipidome of BC patients, and these perturbations in small endogenous molecules are associated with the progression and metastatic potential of BC [30, 31]. Additionally, there is growing evidence supporting that multiple-marker biosignatures are inherently more robust and reliable in diverse clinical settings compared with single biomarkers [32]. Machine learning has also emerged as a pivotal tool in the research pipeline for biomarker discovery and validation [33]. Conventional statistical methods adequately characterize population interferences from a sample. On the other hand, machine learning can recognize potential predictive patterns [34]. Therefore, machine learning can empower exploratory omics-based biomarker studies for human diseases. In this study, we employed a multimodal omics data mining approach coupled with machine learning modeling to identify potential markers for early-stage BC detection.

Seven biomarkers for differentiating cancer and benign were confirmed by the validation process. These biomarkers exhibited outstanding performance in both the linear SVM and random forest models, with AUC values exceeding 0.9. This suggests significant promise of plasma metabolites in aiding the early-stage screening and diagnosis of BC. Among these, certain hydrophilic metabolites such as glutamate and glycochenodeoxycholate were altered in BC patients. For instance, glutamate levels were elevated in the cancer group compared with the benign group. Past research has indicated that accumulation of glutamate plays a pivotal role in energy provision, promotion of signaling pathways, and progression of tumors [35]. The elevated glutamate levels observed in our study may be partly attributed to the dysregulation of glutamine metabolism in cancer cells, particularly enhancement of glutaminolysis, which converts glutamine to glutamate [36]. Furthermore, we observed upregulation of glycochenodeoxycholate levels in BC patients relative to benign patients. This finding aligns with a previous study that reported increased bile acid concentrations in the serum of BC patients compared with healthy controls [37]. Collectively, our findings cohere with earlier reports on metabolic biomarkers in BC, supporting their potential role in BC screening and diagnosis.

The association between lipidomic alterations and BC invasiveness was evident in our study. Notably, PC(O-) exhibited significant differences between the invasive ductal carcinoma and benign tumor groups. The roles of PC(O-) in cancer have been well-documented [38]. It has been reported that ether lipids are involved in membrane trafficking and cell signalling, and are enriched in cancer cells [39]. For instance, some PC(O-) species have been linked to metabolic pathways that provide energy for cancer progression and activate oncogenic signaling pathways, promoting tumor growth [40]. Furthermore, interventional studies on human have suggested that increased circulated ether-linked phosphatidylcholine level could be a predictive biomarker for the progression of prostate cancer or colorectal cancer [41, 42]. Circulating lipid profiles also associated with treatment resistance in prostate cancer, further implicate the important role of lipids in cancer pathophysiology [43]. However, the univariate ROC analysis using the validation set showed subpar performance of PC(O-) compared with SM, which demonstrated better predictive ability. The type of mammography employed in the validation set could be related to this discrepancy. SM has been reported to participate in several intrinsic and extrinsic pathways that mediated cell proliferation and apoptosis via regulation of SM and ceramide balance [44, 45]. Of note, imbalance of SM and Ceramide could cause abnormal apoptotic activity that led to BC cell proliferation [46]. Additionally, alterations in SM metabolism have been correlated with tumor growth and drug resistance [47]. PC and SM lipid subclasses were altered in the plasma of BC patients compared with healthy controls [39]. It is important to note that the exact lipid species were not readily matched between the previous report and our study, possibly due to the difference of the study subjects, i.e., benign tumors rather than healthy controls. Moreover, we detected alterations in the plasma levels of other lipid subclasses, including triglycerides and free fatty acids. Changes in plasma TG levels have been detected in BC [48] and different stages of colorectal cancer [49].

This study has several limitations. First, the sample size was small, and various molecular subtypes were included within each group of interest. However, our focus was on early-stage BC profiles, which are critical for enhancing patient outcomes. The generalizability of our findings may be limited due to the small sample size and high heterogeneity of the molecular subtypes of BC patients. We tried to minimize the over-optimistic validation results by further filtering biomarker candidates (derived from the training set) using the concordance examination and the results of the univariate ROC analysis in the validation set. Then, only several of the most promising biomarker candidates, ranked based on the AUC of the ROC curve, were used for the machine learning model training and cross-validation in the validation set. However, the feature selection procedure was not conducted as part of the machine learning model training process, and the models were established and evaluated on the validation set; it is potentially subjected to selection bias [50, 51]. Second, the study was limited to one BC subtype, specifically invasive ductal carcinoma. This limits the generalizability of our findings to other BC subtypes. Third, the study only assessed relative changes in biomarker levels between cancer and benign patients, without providing quantitative measurements. The implementation of quantitative bioassays is necessary for a more comprehensive validation of the candidate biomarkers. Fourth, there was heterogeneity regarding metastatic status and neoadjuvant chemotherapy between the training and the validation cohort, which may affect the metabolome and lipidome of included patients. Subsequent studies are needed to validate our findings. Fifth, our study compared the early-stage BC patients to individuals with non-malignant tumors. The two groups had highly overlapping clinical manifestations, resulting in subtle differences in plasma metabolic profiles. Only one biomarker detected in the metabolomics analysis remained statistically significant after p-value adjustment. Therefore, pathway analysis for further biological interpretation was not conducted.

## Conclusion

In conclusion, this study employed untargeted metabolomics and lipidomics analyses, coupled with robust feature selection and machine learning modeling, to identify and partially validate potential biomarkers aiding the screening and diagnosis of BC. While many studies on BC typically compared patients of all stages to healthy controls, our study differentiated patient samples to analyze differences between invasive BC and benign tumors. Furthermore, this study offers insights into alterations in hydrophilic and hydrophobic metabolites associated with BC, with a particular focus on the roles of PC(O-) and SM. Future research is imperative to thoroughly validate these biomarkers and to develop robust assay methods. Such biomarkers could serve as valuable tools to enhance screening and diagnosis of BC.

## Supporting information

S1 File Supplementary information of the study. (DOCX)

S2 File (DOCX)

S3 File (DOCX)

S1 Data (XLSX)

S1 Checklist Human participants research checklist. (DOCX)
