# Supplementary material for: Combining metabolomics and machine learning to discover biomarkers for early-stage breast cancer diagnosis
Source: PLoS One. 2024 Oct 21;19(10):e0311810. doi: 10.1371/journal.pone.0311810 (PMC11493280; doi:10.1371/journal.pone.0311810)
Supplement: S2 File — (DOCX) [file pone.0311810.s002.docx]

**Combining metabolomics and machine learning to discover biomarkers for early-stage breast cancer diagnosis**

Nguyen Ky Anh^1,2,¶^, Anbok Lee^3,¶^, Nguyen Ky Phat^2^, Nguyen Thi Hai Yen^2^, Nguyen Quang Thu^2^, Nguyen Tran Nam Tien^2^, Ho-Sook Kim^2^, Tae Hyun Kim^4^, Dong Hyun Kim^2^, Hee-Yeon Kim^4,*^, Nguyen Phuoc Long^2,*^

^1^Faculty of Pharmacy, Ton Duc Thang University, Ho Chi Minh City, Vietnam

^2^Department of Pharmacology and PharmacoGenomics Research Center, Inje University College of Medicine, Busan, Republic of Korea

^3^Department of Surgery, Chung-Ang University Gwang-Myeong Hospital, Chung-Ang University College of Medicine, Gyeonggi-do, Republic of Korea

^4^Department of Surgery, Busan Paik Hospital, Inje University College of Medicine, Busan, Republic of Korea

**^*^**Corresponding authors: Hee-Yeon Kim and Nguyen Phuoc Long.

Email: [gsrcq@naver.com](mailto:gsrcq@naver.com) (HYK), [phuoclong@inje.ac.kr](mailto:phuoclong@inje.ac.kr) (NPL).

^¶^: These authors contributed equally to this work.

**SUPPLEMENTARY FIGURES**

**S1 Fig. Principal component analysis scores plots of metabolomics and lipidomics data with quality control samples.** A. Metabolomics positive ion mode. B. Metabolomics negative ion mode. C. Lipidomics positive ion mode. D. Lipidomics negative ion mode.


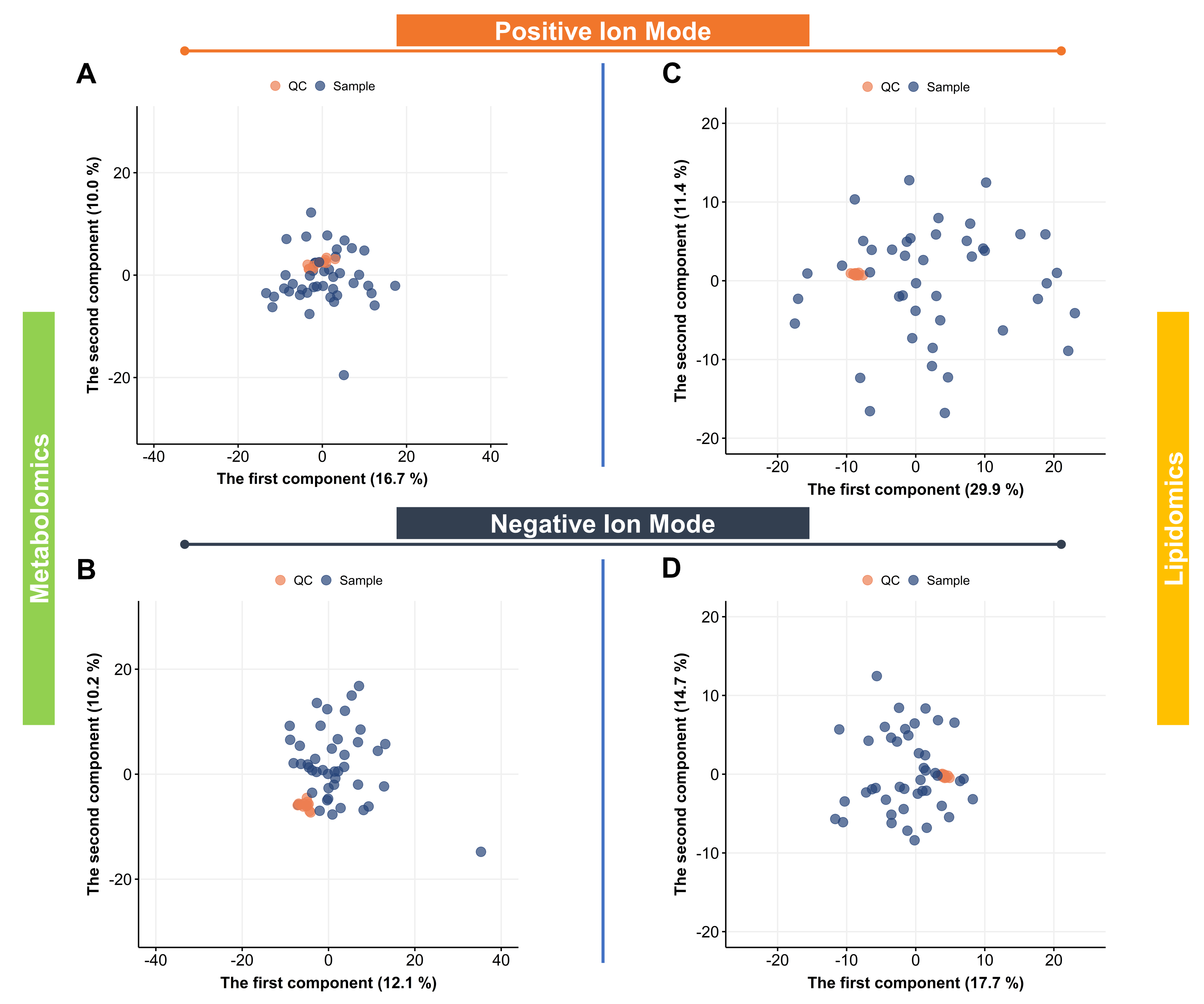


**S2 Fig. Concordance examination for the tentative biomarker levels in breast cancer and benign tumor controls.** The dark blue circle indicates the metabolites with inconsistent levels between the training and the validation sets. Cer, Ceramide; DG, Diacylglycerol; FA, Free fatty acid; HexCer, Hexosylceramide; Hex2Cer, Dihexosylceramide; LPC, Lysophosphatidylcholine; LPC(O-), Ether-linked lysophosphatidylcholine; LPE, Lysophosphatidylethanolamine; PC, Phosphatidylcholine; PC (O-), Ether-linked phosphatidylcholine; PE (O-), Ether-linked phosphatidylethanolamine; PI, Phosphatidylinositol; SM, Sphingomyelin; TG, triacylglycerol.

**
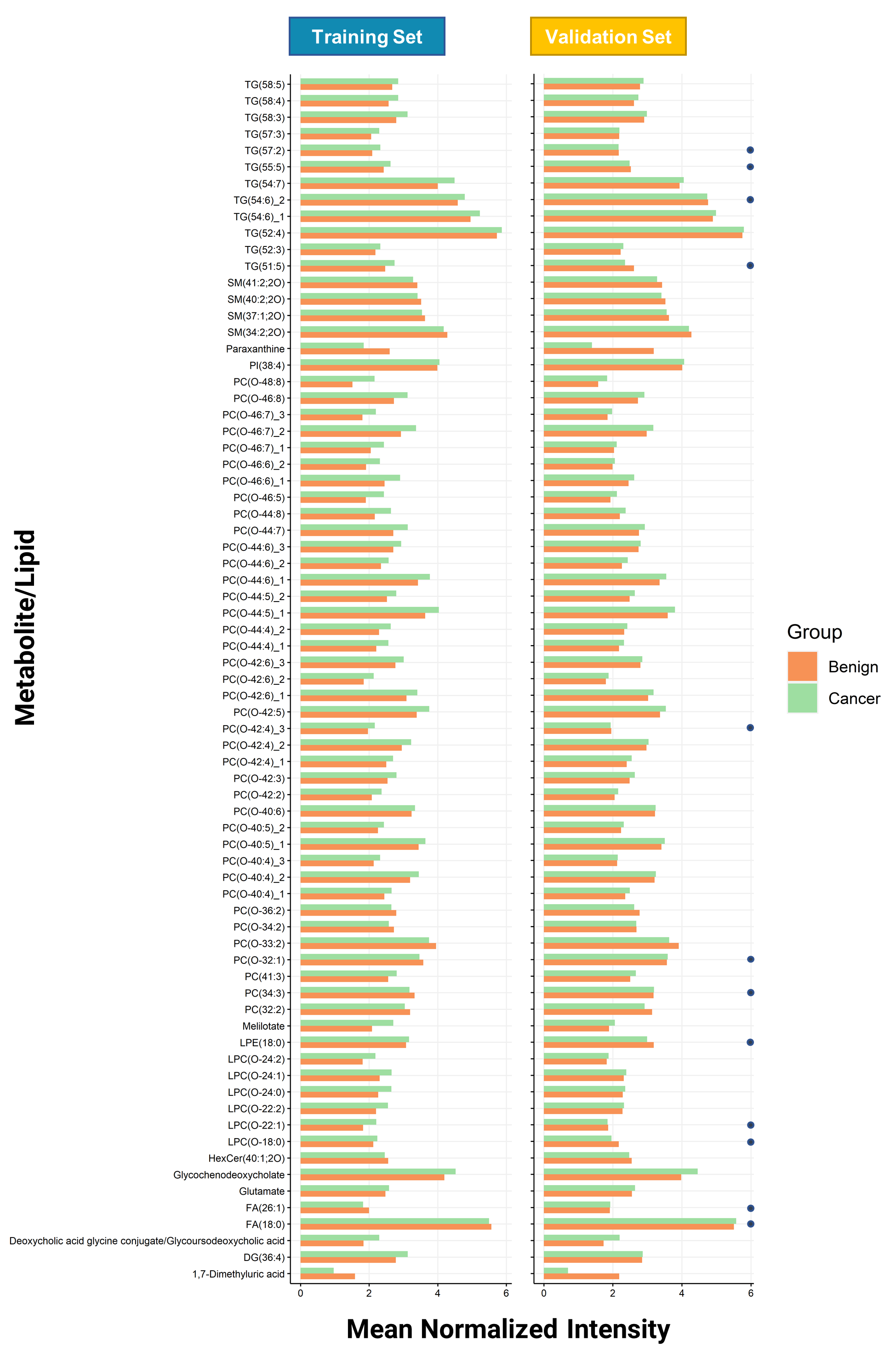
**

**SUPPLEMENTARY TABLES**

**S1 Table. Statistical descriptions of the biomarker candidates.**

| ID | Analyte | Ion mode | Regulation in BC | Linear model result | | | Univariate ROC analysis result | |
| --- | --- | --- | --- | --- | --- | --- | --- | --- |
|  |  |  |  | Fold change | p-value | FDR | AUC | p-value |
| Analytes detected in metabolomics | | | | | | | | |
| 1 | Deoxycholic acid glycine conjugate/Glycoursodeoxycholic acid | Positive | Up | - | - | - | 0.829 | 1.46E-03 |
| 2 | LPC(O-22:2) | Positive | Up | 2.302 | 1.93E-04 | 2.01E-01 | 0.808 | 2.05E-04 |
| 3 | Dimethyluric acid | Negative | Down | - | - | - | 0.739 | 2.76E-02 |
| 4 | Glutamate | Negative | Up | - | - | - | 0.726 | 4.45E-02 |
| 5 | Glycochenodeoxycholate | Negative | Up | - | - | - | 0.701 | 3.10E-02 |
| 6 | LPE(18:0) | Negative | Up | - | - | - | 0.739 | 4.08E-02 |
| 7 | Melilotate | Negative | Up | - | - | - | 0.731 | 1.15E-02 |
| 8 | Paraxanthine | Negative | Down | - | - | - | 0.761 | 2.03E-02 |
| Analytes detected in lipidomics | | | | | | | | |
| 9 | DG(36:4) | Positive | Up | - | - | - | 0.735 | 2.26E-02 |
| 10 | LPC(O-18:0) | Positive | Up | - | - | - | 0.739 | 4.82E-02 |
| 11 | LPC(O-22:1) | Positive | Up | 2.523 | 2.46E-04 | 2.47E-02 | 0.803 | 2.94E-04 |
| 12 | LPC(O-24:0)^1^ | Positive | Up | 2.326 | 2.49E-05 | 1.17E-02 | 0.897 | 6.41E-06 |
| 13 | LPC(O-24:1) | Positive | Up | 2.174 | 3.84E-04 | 3.23E-02 | 0.825 | 1.76E-04 |
| 14 | LPC(O-24:2) | Positive | Up | 2.324 | 1.57E-04 | 2.22E-02 | 0.838 | 6.62E-05 |
| 15 | PC(41:3) | Positive | Up | 1.707 | 4.88E-03 | 1.68E-01 | 0.769 | 2.73E-03 |
| 16 | PC(O-32:1) | Positive | Down | - | - | - | 0.748 | 1.36E-02 |
| 17 | PC(O-33:2) | Positive | Down | - | - | - | 0.709 | 4.53E-02 |
| 18 | PC(O-36:2) | Positive | Down | - | - | - | 0.726 | 2.23E-02 |
| 19 | PC(O-40:4)^2^ | Positive | Up | 1.905 | 1.51E-03 | 7.12E-02 | 0.752 | 3.33E-03 |
| 20 | PC(O-40:4) | Positive | Up | 1.593 | 4.51E-03 | 1.63E-01 | 0.761 | 2.93E-03 |
| 21 | PC(O-40:5)^3^ | Positive | Up | - | - | - | 0.718 | 1.13E-02 |
| 22 | PC(O-40:6) | Positive | Up | - | - | - | 0.735 | 2.65E-02 |
| 23 | PC(O-42:2) | Positive | Up | 1.849 | 2.01E-04 | 2.42E-02 | 0.902 | 6.12E-05 |
| 24 | PC(O-42:3) | Positive | Up | 1.796 | 4.29E-03 | 1.59E-01 | 0.748 | 3.37E-03 |
| 25 | PC(O-42:4) | Positive | Up | 1.941 | 7.58E-04 | 4.38E-02 | 0.782 | 9.44E-04 |
| 26 | PC(O-42:4) | Positive | Up | - | - | - | 0.739 | 8.21E-03 |
| 27 | PC(O-42:5) | Positive | Up | 2.525 | 8.08E-04 | 4.38E-02 | 0.774 | 1.91E-03 |
| 28 | PC(O-42:5) | Positive | Up | 2.001 | 4.77E-03 | 1.68E-01 | 0.744 | 1.18E-02 |
| 29 | PC(O-42:6)^4^ | Positive | Up | 2.135 | 4.70E-04 | 3.69E-02 | 0.791 | 5.62E-04 |
| 30 | PC(O-42:6) | Positive | Up | 1.767 | 1.03E-03 | 5.03E-02 | 0.756 | 6.97E-04 |
| 31 | PC(O-44:4) | Positive | Up | 2.202 | 2.06E-04 | 2.42E-02 | 0.829 | 1.12E-04 |
| 32 | PC(O-44:4) | Positive | Up | 2.225 | 1.99E-03 | 8.77E-02 | 0.778 | 1.64E-03 |
| 33 | PC(O-44:5) | Positive | Up | 2.653 | 7.94E-04 | 4.38E-02 | 0.769 | 1.37E-03 |
| 34 | PC(O-44:6) | Positive | Up | 2.281 | 5.67E-04 | 3.91E-02 | 0.782 | 6.14E-04 |
| 35 | PC(O-44:6) | Positive | Up | 1.701 | 2.49E-03 | 1.06E-01 | 0.765 | 1.41E-03 |
| 36 | PC(O-44:7) | Positive | Up | 2.600 | 9.56E-05 | 1.88E-02 | 0.876 | 4.41E-05 |
| 37 | PC(O-44:7) | Positive | Up | 2.096 | 1.03E-03 | 5.03E-02 | 0.825 | 2.86E-04 |
| 38 | PC(O-44:8) | Positive | Up | 2.704 | 1.20E-04 | 1.88E-02 | 0.889 | 3.26E-05 |
| 39 | PC(O-46:5) | Positive | Up | 3.297 | 7.27E-05 | 1.80E-02 | 0.859 | 3.75E-05 |
| 40 | PC(O-46:6) | Positive | Up | 2.735 | 7.17E-05 | 1.80E-02 | 0.846 | 2.75E-05 |
| 41 | PC(O-46:6) | Positive | Up | 2.336 | 3.34E-04 | 3.14E-02 | 0.855 | 9.04E-05 |
| 42 | PC(O-46:7) | Positive | Up | 2.230 | 7.66E-05 | 1.80E-02 | 0.876 | 1.72E-05 |
| 43 | PC(O-46:7)^5^ | Positive | Up | 2.638 | 2.41E-04 | 2.47E-02 | 0.863 | 9.26E-05 |
| 44 | PC(O-46:8) | Positive | Up | 2.332 | 1.15E-04 | 1.88E-02 | 0.889 | 2.83E-05 |
| 45 | PC(O-48:8) | Positive | Up | 3.760 | 1.28E-05 | 1.02E-02 | 0.910 | 4.04E-06 |
| 46 | PI(38:4) | Positive | Up | - | - | - | 0.735 | 3.03E-02 |
| 47 | SM(37:1;2O) | Positive | Down | - | - | - | 0.774 | 1.75E-02 |
| 48 | SM(42:4;3O) | Positive | Up | 1.612 | 3.24E-03 | 1.30E-01 | 0.774 | 2.22E-03 |
| 49 | TG(51:5) | Positive | Up | - | - | - | 0.748 | 1.81E-02 |
| 50 | TG(52:3) | Positive | Up | - | - | - | 0.726 | 2.22E-02 |
| 51 | TG(52:4) | Positive | Up | - | - | - | 0.731 | 3.14E-02 |
| 52 | TG(54:6) | Positive | Up | - | - | - | 0.726 | 2.72E-02 |
| 53 | TG(54:6) | Positive | Up | - | - | - | 0.735 | 3.98E-02 |
| 54 | TG(54:7) | Positive | Up | - | - | - | 0.752 | 2.42E-02 |
| 55 | TG(55:5) | Positive | Up | - | - | - | 0.714 | 1.67E-02 |
| 56 | TG(57:2) | Positive | Up | - | - | - | 0.726 | 4.04E-02 |
| 57 | TG(57:3) | Positive | Up | - | - | - | 0.722 | 2.77E-02 |
| 58 | TG(58:3) | Positive | Up | - | - | - | 0.714 | 3.86E-02 |
| 59 | TG(58:4) | Positive | Up | - | - | - | 0.722 | 3.87E-02 |
| 60 | TG(58:5) | Positive | Up | - | - | - | 0.726 | 3.92E-02 |
| 61 | TG(60:4) | Positive | Up | - | - | - | 0.726 | 2.43E-02 |
| 62 | FA(18:0) | Negative | Down | - | - | - | 0.709 | 4.37E-02 |
| 63 | FA(26:1) | Negative | Down | 0.713 | 1.12E-02 | 2.31E-01 | 0.825 | 3.19E-03 |
| 64 | HexCer(40:1;2O) | Negative | Down | - | - | - | 0.722 | 1.39E-02 |
| 65 | LPC(O-24:0)^1^ | Negative | Up | 2.693 | 4.88E-05 | 3.08E-02 | 0.885 | 1.81E-05 |
| 66 | PC(32:2) | Negative | Down | - | - | - | 0.726 | 4.91E-02 |
| 67 | PC(34:3) | Negative | Down | - | - | - | 0.731 | 2.74E-02 |
| 68 | PC(O-34:2) | Negative | Down | 0.697 | 8.04E-04 | 4.20E-02 | 0.833 | 2.78E-03 |
| 69 | PC(O-40:4)^2^ | Negative | Up | 1.658 | 8.41E-04 | 4.20E-02 | 0.778 | 3.85E-03 |
| 70 | PC(O-40:5)^3^ | Negative | Up | 1.546 | 2.68E-03 | 9.15E-02 | 0.752 | 3.96E-03 |
| 71 | PC(O-42:4) | Negative | Up | 1.680 | 4.15E-03 | 1.18E-01 | 0.748 | 8.49E-03 |
| 72 | PC(O-42:6)^4^ | Negative | Up | 2.021 | 6.67E-04 | 4.20E-02 | 0.821 | 8.66E-04 |
| 73 | PC(O-44:5) | Negative | Up | 1.970 | 5.91E-04 | 4.20E-02 | 0.769 | 1.18E-03 |
| 74 | PC(O-44:6) | Negative | Up | 1.734 | 7.01E-04 | 4.20E-02 | 0.795 | 1.06E-03 |
| 75 | PC(O-46:7)^5^ | Negative | Up | 2.396 | 1.75E-04 | 3.08E-02 | 0.859 | 7.75E-05 |
| 76 | SM(34:2;2O) | Negative | Down | - | - | - | 0.726 | 1.55E-02 |
| 77 | SM(40:2;2O) | Negative | Down | - | - | - | 0.761 | 1.24E-02 |
| 78 | SM(41:2;2O) | Negative | Down | 0.730 | 7.34E-03 | 1.79E-01 | 0.769 | 1.07E-02 |
| Abbreviations: BC, Breast Cancer; AUC, Area Under the Curve; FA, Free fatty acid; HexCer, Hexosylceramide; LPC(O-), Ether-linked Lysophosphatidylcholine; LPE, Lysophosphatidylethanolamine; PC, Phosphatidylcholine; PC(O-), Ether-linked Phosphatidylcholine; PI, Phosphatidylinositol; SM, Sphingomyelin; DG, Diacylglycerol; TG, Triacylglycerol.  ^1-5^: Analytes detected in both positive and negative modes. | | | | | | | | |
