# Supplementary material for: Combining metabolomics and machine learning to discover biomarkers for early-stage breast cancer diagnosis
Source: PLoS One. 2024 Oct 21;19(10):e0311810. doi: 10.1371/journal.pone.0311810 (PMC11493280; doi:10.1371/journal.pone.0311810)
Supplement: S3 File — (DOCX) [file pone.0311810.s003.docx]

**Combining metabolomics and machine learning to discover biomarkers for early-stage breast cancer diagnosis**

1. **Required Statements**

Select All.

1. **United States Government Employee**

No – No authors are employees of US Government.

1. **Financial Disclosure**

Yes.

**Funding details:**

This work was supported by 2022 Inje University Busan Paik Hospital Research Grant. The funding organization did not participate in the study design, data acquisition, data analysis and interpretation, or the content of the manuscript.

**Country of your main research funder**

KOREA, REPUBLIC OF

1. **Competing Interests**

The authors declare that they have no known competing financial interests or personal relationships that could have appeared to influence the work reported in this paper.

1. **Ethics Statement**

The study was conducted with the approval of the Institutional Review Board of Inje University College of Medicine Busan Paik Hospital (IRB No. 2022-08-052). All participants provided written informed consent for the use of their clinical information and plasma samples in the study.

1. **Figure Guidelines**

Yes - I confirm our figures comply with the guidelines.

1. **Copyrighted Figures**

Blank

1. **Dual Publication**

No

1. **Previous Interactions**

Clear all

1. **Group Authorship**

No

1. **Collections and Calls for Papers**

Blank

1. **Request Editor**

Blank

1. **New Species**

N/A

1. **Data Availability**

No – some restrictions will apply.

**Describe where the data may be found in full sentences:**

Normalized metabolomics and lipidomics data were shared in the supporting information of the manuscript.

1. **Immediate Preprint Posting**

No

1. **Subsection(s)**

Cancer – General

Systems biology

Women’s and maternal health

1. **Collections**

No

1. **Institutional Account**

No
